# Efficacy and Tolerability of Nintedanib in Idiopathic-Inflammatory-Myopathy-Related Interstitial Lung Disease: A Pilot Study

**DOI:** 10.3389/fmed.2021.626953

**Published:** 2021-02-03

**Authors:** Junyu Liang, Heng Cao, Yang Yang, Yini Ke, Ye Yu, Chuanyin Sun, Lihuan Yue, Jin Lin

**Affiliations:** Department of Rheumatology, The First Affiliated Hospital, Zhejiang University School of Medicine, Hangzhou, China

**Keywords:** nintedanib, interstitial lung disease, dermatomyositis, polymyositis, anti-MDA5 antibody

## Abstract

**Objectives:** To initially clarify the efficacy and tolerability of nintedanib in patients with idiopathic-inflammatory-myopathy-related interstitial lung disease (IIM-ILD).

**Methods:** A retrospective, real-world analysis was conducted in IIM-ILD patients who regularly received outpatient visit or hospitalization from January 2018 to March 2020 in three centers. And the patients were divided into two groups depending on presence or absence of nintedanib therapy. Comparisons, Kaplan-Meier survival analysis and propensity score matching were made to identify difference in time to death from any cause, incidence of rapidly progressive interstitial lung disease (RP-ILD) and comorbidity of pulmonary infection between the two groups. The following logistic regression analyses and Cox proportional-hazard regression analyses were used to verify the therapeutic value of nintedanib as well as clinical significance of other factors. Adverse events were descriptively recorded.

**Results:** Thirty-six patients receiving nintedanib therapy and 115 patients without use of nintedanib were included. Before and after propensity score matching, the primary comparisons revealed better survival (*P* = 0.015, *P* = 0016, respectively) and lower incidence of RP-ILD (*P* = 0.017, *P* = 0.014, respectively) in patients with nintedanib therapy. Logistic regression analysis identified that disease activity (*P* < 0.001), percent-predicted diffusing capacity of the lung for carbon monoxide (DLCO%, *P* = 0.036), nintedanib therapy (*P* = 0.004, OR value = 0.072) and amyopathic dermatomyositis (ADM, *P* = 0.012) were significantly correlated with RP-ILD. Cox proportional hazards regression analysis suggested that disease activity (*P* < 0.001), anti-MDA5 antibody (*P* < 0.001) and nintedanib therapy (*P* = 0.013, HR value=0.268) were significantly associated with survival of IIM-ILD patients. Similar results can also be seen in analyses after propensity score matching. In the 36 patients with nintedanib therapy, diarrhea was the most common adverse event (44.4%) and hepatic insufficiency contributed to most dosage reduction (44.4% of nine patients) or therapy discontinuation (60.0% of five patients).

**Conclusions:** Nintedanib was found to reduce incidence of RP-ILD and improve survival in IIM-ILD patients in a real-world setting. Anti-MDA5 antibody could be taken as a risk factor for unfavorable outcome. ADM was significantly correlated with occurrence of RP-ILD. In addition to the most frequent diarrhea, hepatic insufficiency was closely related to dosage reduction or therapy discontinuation.

## Introduction

Idiopathic inflammatory myopathy (IIM) comprises a group of autoimmune diseases featuring systemic inflammation of the proximal skeletal muscles and/or the skin (1, 2) Dermatomyositis (DM) and polymyositis (PM) are two classical subtypes of IIM, whereas amyopathic dermatomyositis (ADM) is a newly recognized subgroup of DM with typical skin rash of DM and slight/absent muscular impairment (1–3). IIM contributes to elevated morbidity and mortality rate due to severe muscle weakness as well as multiple organ involvement including esophagus, heart, and lungs in particular (4). Interstitial lung disease (ILD) is the most common extra-muscular target in IIM patients, with a reported prevalence of 78%, and is found in 65% of newly diagnosed IIM cases (5–7). Idiopathic-inflammatory-myopathy-related ILD (IIM-ILD) is frequently aggressive and refractory to conventional therapies including glucocorticoids and immunosuppressive drugs (7–9). In different studies, the incidences of rapidly progressive interstitial lung disease (RP-ILD) in IIM patients were relatively high (24–8%) compared to those in idiopathic pulmonary fibrosis (IPF, 8.6–23.9%) and systemic-sclerosis-related ILD (Ssc-ILD, 9.4%) (10–13). RP-ILD has been taken as a major cause of mortality in IIM patients (14, 15). To alleviate lung involvement, immunosuppressive medications could be potent and sometimes costly, which also made the patients vulnerable to infection (16). Under such circumstance, it would be necessary to take a broader view and search for more potent therapeutic agent for IIM-ILD.

Nintedanib, formerly known as BIBF 1120, has been recognized as an effective antifibrotic agent (17–19). Preceding research in animal models with manifestations of fibrosing ILDs like IPF, Ssc-ILD, rheumatoid-arthritis-related ILD (RA-ILD), hypersensitivity pneumonitis and silicosis has uncovered that nintedanib has anti-fibrotic effect regardless of the trigger for the ILD pathology (20). Furthermore, the therapeutic value of nintedanib has been suggested in IPF and Ssc-ILD *via* TOMORROW, INPULSIS-1, INPULSIS-2 trials as well as several real-world analyses (21–23). The SENSCIS trial proved the efficacy of nintedanib in Ssc-ILD with a majority of non-UIP pattern (24, 25). The most recent INBUILD trial suggested that nintedanib reduced the rate of ILD progression in patients with chronic fibrosing ILD and progressive phenotype, including autoimmune ILD with usual interstitial pneumonia (UIP) or non-UIP pattern (26). However, the efficacy and safety of nintedanib in the subtype of IIM-ILD remain unclear. The unfavorable outcome, potent immunosuppressive therapies, the frequent comorbidities of RP-ILD and infection precipitated us to seek to introduce nintedanib into therapy of IIM-ILD.

Encouraged by the initiation of INBUILD and SENSCIS trials, after the approval (2018-224) from the Institutional Review Board (IRB) of the First Affiliated Hospital, Zhejiang University School of Medicine (FAHZJU), combined use of nintedanib and immunosuppressive medications was initiated for a few IIM-ILD patients after clarifying diagnosis of IIM-ILD and acquiring written informed consent. Other IIM-ILD patients who declined in the same period received conventional immunosuppressive therapies. The evaluation and inqury were implemented for all IIM-ILD patients in the outpatient and inpatient department of Qingchun, Chengzhan and Zhijiang divisions of FAHZJU. To acquire an initial understanding on the efficacy and tolerability of nintedanib in IIM-ILD, we reviewed the records of all IIM-ILD patients who were regularly treated and followed-up in three divisions from January 2018 to March 2020, and performed a real-world analysis to evaluate the therapeutic value, dosage regimen and profile of adverse events of nintedanib in IIM-ILD.

## Patients and Methods

### Patients

After acquiring the approval (Reference Number: 2020-200, 2018-224) from the IRB of FAHZJU and written informed consent to utilize and publish clinical data from all patients involved, in accordance with the Declaration of Helsinki, we retrieved medical records of adult patients who were regularly treated and visited in the outpatient or inpatient department of Qingchun, Chengzhan and Zhijiang divisions of FAHZJU with the diagnosis of IIM-ILD from January 2018 to March 2020. The inclusion criteria of this study were: (1) age over 18 years old; (2) the definite/probable diagnosis of DM, PM or ADM satisfied the 2017 ACR/EULAR classification criteria (27); (3) ILD on high-resolution computed tomography (HRCT) at their first outpatient visit or within the first week of admission, including UIP patterns and non-UIP patterns (non-specific interstitial pneumonia, cryptogenic organizing pneumonia, co-existence of more than one CT pattern), as confirmed by experienced radiologists; (4) regular outpatient visit or hospitalization in FAHZJU; Exclusion criteria were: (1) clarified overlap syndromes with other connective tissue diseases (CTDs); (2) outpatient visit or hospitalization for reasons unrelated to myositis and its comorbidities, such as fracture, pregnancy, acquired immunodeficiency syndrome and cataract, etc. due to lack of demanded medical records for this study; (3) preceding use of nintedanib, or preceding/present use of pirfenidone; (4) loss to follow-up without death from any cause within 6 months after initial outpatient visit or hospitalization. The included patients were divided into nintedanib group and control group depending on the presence or absence of nintedanib therapy.

### Methods

Medical records of all enrolled patients were retrospectively collected by reviewing the electronic medical record (EMR) system of FAHZJU. Data including demographic information, disease activity assessment, clinical manifestations or comorbidities, preceding comorbidities, harmful hobbies, radiological/laboratory findings, lung function testing, immunosuppressive medications as well as use of nintedanib were acquired and analyzed. To obtain the survival, incidence of RP-ILD and pulmonary infection in all the patients involved, patients were followed from the initial outpatient visit or hospitalization until the end of follow-up. For patients who perished during hospitalization, their dates of death were clearly documented in the EMR system. For patients who were discharged, a routine return visit was arranged 2 weeks after discharge. In addition to the regular inpatient or outpatient visits, a concise telephone interview was performed 3 months after discharge, and at an annual frequency afterwards. The end of follow-up could be owing to death from any cause, loss to follow-up, or closure of follow-up for the purpose of this study (September 30th, 2020). In patients receiving nintedanib therapy, initial dosage, dosage adjustment, discontinuation and duration of nintedanib as well as adverse events were also collected from the EMR and follow-up records. Time to death from any cause and occurrence of RP-ILD in the follow-up were our major concerns. Since comorbidities and progression of ILD usually lead to more potent immunosuppressive therapy and the subsequent comorbidity of pulmonary infection in clinical practice, comorbidity of pulmonary infection was also taken into account. Due to the initial lack of follow-up lung function testing and recent impact of Corona virus disease 2019 (COVID-19) pandemic, decline of forced vital capacity (FVC) was not listed as an end point in this study. Adverse events mainly incorporated diarrhea, abdominal pain, nausea and vomiting, anorexia, weight loss, fatigue, hepatic insufficiency and cough.

Disease activity of IIM was routinely assessed utilizing the Myositis Disease Activity Assessment Visual Analog Scales (MYOACT) (28). Baseline disease activity assessment, lung function testing and HRCT scan were performed at the initial outpatient visit or within the first week of hospitalization. ILD and its progression were evaluated by experienced radiologists using HRCT. Cases manifested as definite or probable UIP pattern were recognized based on their HRCT appearance: the presence of basal-dominant reticular opacities and predominantly basal and subpleural distribution of honeycomb lesions, with multiple equal-sized cystic lesions of 2 to 10 mm diameter with a thick wall (29). A subset of patients with RP-ILD was defined as those presenting with progressive dyspnea and progressive hypoxemia, a worsening of interstitial change on the chest radiograph within 1 month after the initial visit or onset of respiratory symptoms (30–33). Infection, pulmonary infection in particular, was a common comorbidity in IIM patients and a key contributor to unfavorable outcome. Identification of pulmonary infection relied on International Statistical Classification of Diseases, 10th revision (ICD-10)-coded discharge diagnosis of community-acquired pneumonia (CAP), hospital-acquired pneumonia (HAP), pulmonary fungal infection or pulmonary infection. Responsible pathogens were recognized based on repeated cultures/smears of bronchoalveolar lavage fluid (BALF) or sputum before related treatment. To acquire the positivity of 12 myositis-specific antibodies (MSAs, anti-MDA5, anti-TIF1γ, anti-Jo-1, anti-OJ, anti-PL-7, anti-PL-12, anti-EJ, anti-Mi-2α, anti-Mi-2β, anti-NXP2, anti-SRP and anti-SAE1) and 4 myositits-associated antibodies (MAAs, anti-Ro-52, anti-PM-Scl75, anti-PM-Scl100, anti-Ku) of IIM patients (34), sera from these patients were acquired at their first outpatient visit or within the first week of admission. The samples were tested using the EUROLINE Autoimmune Inflammatory Myopathies 16 Ag (IgG) commercial line blot assay (Euroimmun, Lübeck, Germany) encompassing a membrane strip with the 16 autoantigens in light of the manufacturer's instructions.

### Statistical Analysis

Statistical analysis was performed using SPSS 22.0 (Chicago, IL, USA) and R 3.6.1. In comparison between patients receiving nintedanib therapy and patients under conventional medications, independent sample *t*-test was used to compare normally distributed continuous variables. Mann-Whitney *U*-test was applied to compare skewed continuous variables or ordinal categorical variables. Chi-square test and Fisher's exact-test were utilized to compare unordered categorical variables. Propensity score matching was performed to adjust for age, sex as well as statistically different factors in comparison. The propensity scores were calculated using logistic regression analysis with Statistical Package for SPSS 22.0 (Chicago, IL, USA). To be specific, a 1:3 matching was performed without replacement, using nearest neighbor matching and a caliper width of 0.5 of the standard deviation. Survival in groups with nintedanib therapy or conventional treatment was estimated using the Kaplan-Meier method with log-rank-test. Cox proportional hazards regression analyses were subsequently adopted to identify the influence of nintedanib and other factors on the time to death from any cause. Logistic regression analyses were used to unveil whether nintedanib and other factors impacted the left end point (the risk of RP-ILD, comorbidity of pulmonary infection) with statistical significance. Explanatory factors with *P* < 0.05 in the univariate analyses would be entered into the multivariate analyses. All tests were two-sided, and *P* < 0.05 was considered statistically significant. Adverse events were presented descriptively.

## Results

**Figure 1:**
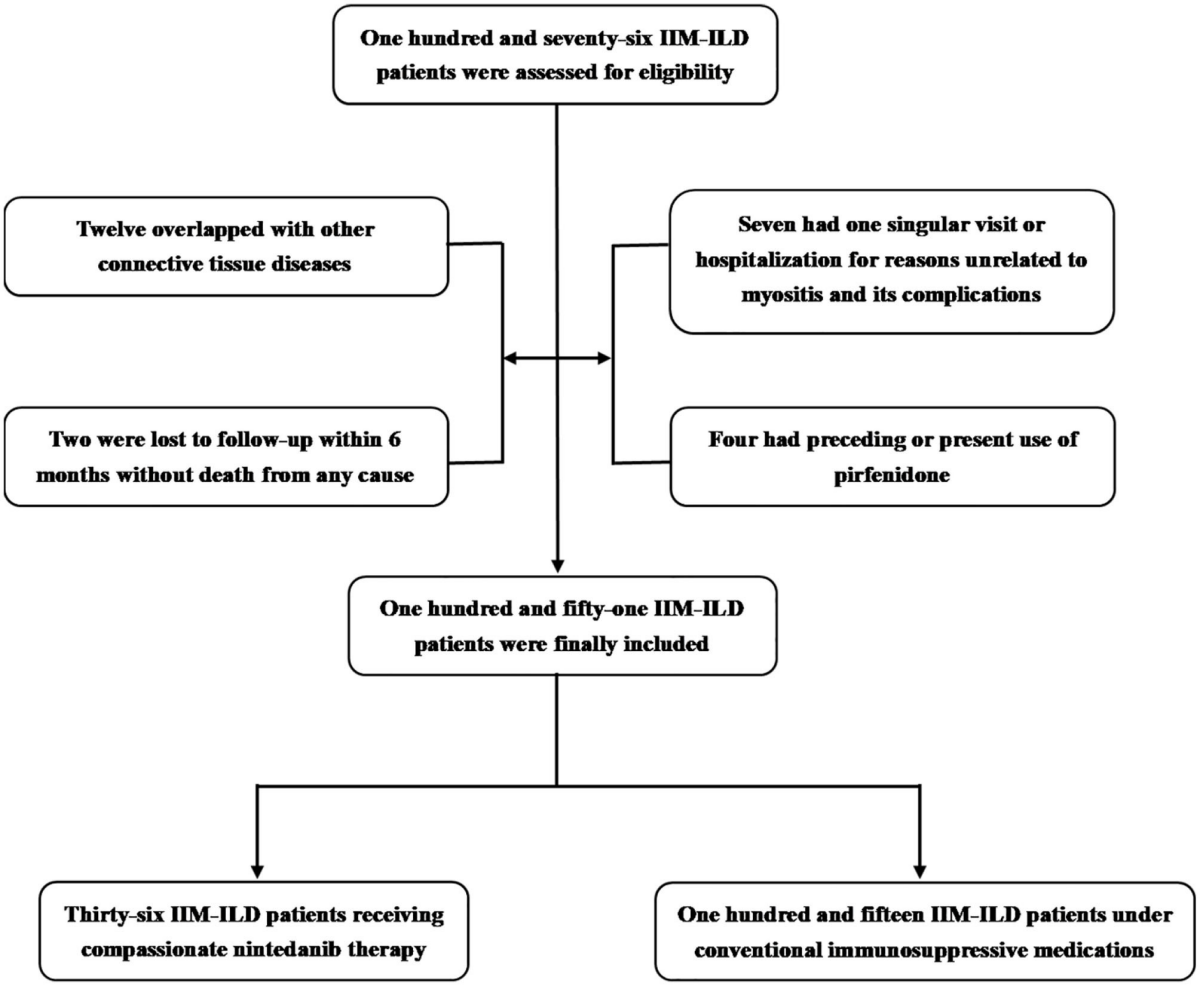
Enrollment and groupings of this study. IIM-ILD, Idiopathic-inflammatory-myopathy-related interstitial lung disease.

A total of 151 IIM-ILD patients who satisfied the inclusion and exclusion criteria from January 2018 to March 2020 were finally incorporated into this study (Figure 1), encompassing 96 with DM, 36 with PM and 19 with ADM. 55 were males (36.4%) and the mean age of all the patients included was 56.3 ± 11.5 years old. The medium follow-up time was 11.7 (6.3, 17.0) months. For the 36 patients who agreed and signed written informed consent on nintedanib therapy (Supplementary Table 1), nintedanib was initially administered 150 mg twice daily as per the manufacturer's recommendation. But dosage reduction or discontinuation of nintedanib therapy would occur due to adverse events. The immunosuppressive regimens included: (1) systemic prednisolone (PSL), methylprednisolone (mPSL) with a maximum dosage ≥ 1 mg/kg/d (calculated by prednisolone); (2) combined therapy of PSL/mPSL, disease-modifying anti-rheumatic drugs (DMARDs) and/or intravenous immunoglobulin (IVIG). And the DMARDs used in this study included Mycophenolate, Thalidomide, Hydroxychloroquine, Cyclosporine, Methotrexate, Cyclophosphamide. Among the 36 patients, 19.4% received potent steroid monotherapy, and 80.6% received combined therapy of steroid and DMARDs/IVIG. The other 115 patients (control group) who declined nintedaniib therapy were under conventional immunosuppressive medications, including 24.3% receiving potent steroid monotherapy and 75.7% receiving combined therapy of steroid and DMARDs/IVIG. 5.6% of patients with nintedanib therapy developed RP-ILD, in comparison to 23.5% in the control group.

**Figure 2:**
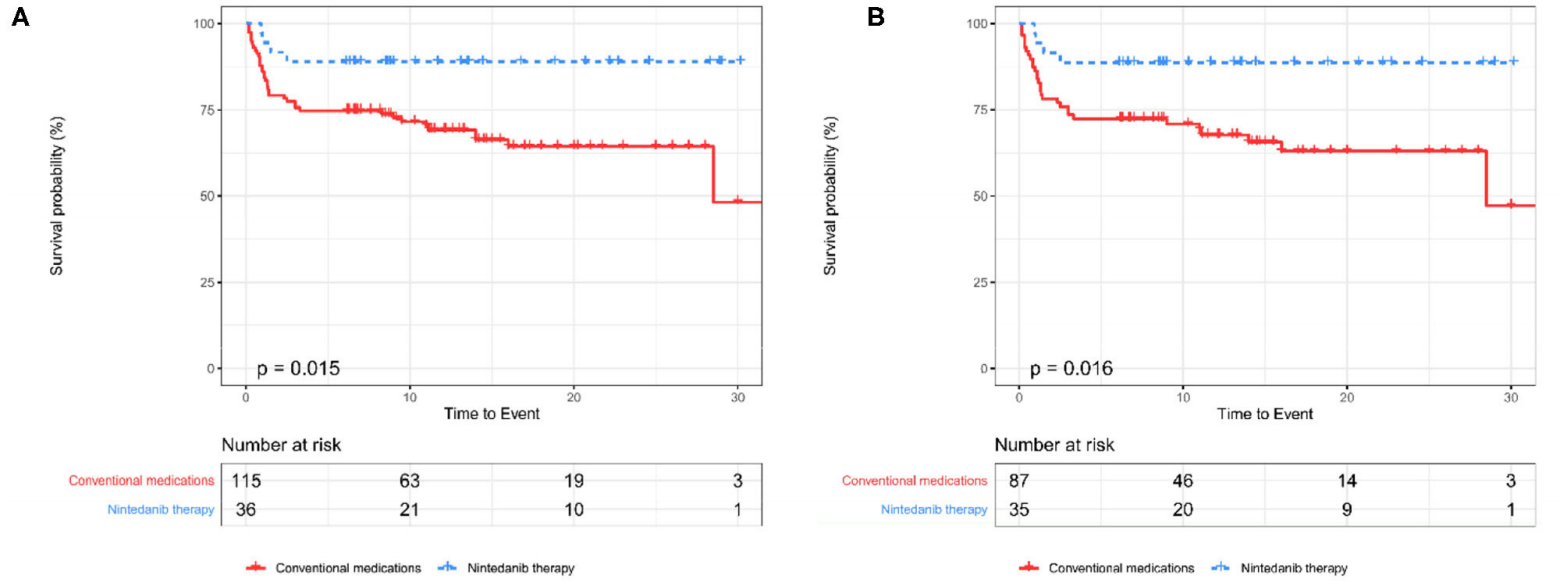
The Kaplan-Meier curves between patients receiving nintedanib therapy and patients under conventional medications before and after PSM **(A,B)**. PSM, Propensity score matching.

An unadjusted comparison between patients receiving nintedanib therapy and patients under conventional medications identified that patients with nintedanib therapy had lower percent-predicted diffusing capacity of the lung for carbon monoxide (DLCO%, *P* = 0.049) and more comorbidity of hypertension (*P* = 0.038), but exhibited better survival (*P* = 0.015, Figure 2) as well as lower incidence of RP-ILD (*P* = 0.017). After adjusting for age, sex, DLCO% and hypertension using propensity score matching, better survival (*P* = 0.016, Figure 2) and lower incidence of RP-ILD (*P* = 0.014) could still be identified in patients receiving nintedanib therapy. No statistical significance was found in comorbidity of pulmonary infection between the two groups (Table 1).

**Table 1:** Unadjusted comparisons between patients receiving nintedanib therapy and patients under conventional medications.

**Factors**	**Before propensity score matching**			**After propensity score matching**		
	**Nintedanib (36)**	**Non-nintedanib (115)**	***P*-value**	**Nintedanib (35)**	**Non-nintedanib (87)**	***P*-value**
Age (y)	57.3 ± 10.0	56.0 ± 11.9	0.529	57.1 ± 10.1	56.5 ± 12.3	0.788
Sex (male/female)	14/22	41/74	0.725	14/21	30/57	0.566
**Clinical manifestations or comorbidities**						
Dysphagia	4 (11.1%)	19 (16.5%)	0.430	3 (8.6%)	12 (13.8%)	0.624
Dysarthria	1 (2.8%)	9 (7.8%)	0.497	1 (2.9%)	5 (5.7%)	0.672
Respiratory muscle involvement	0 (0.0%)	5 (4.3%)	0.339	0 (0.0%)	2 (2.3%)	1.000
Cardiac involvement	4 (11.1%)	4 (3.5%)	0.174	4 (11.4%)	3 (3.4%)	0.103
Gastrointestinal hemorrhage	4 (11.1%)	6 (5.2%)	0.391	4 (11.4%)	5 (5.7%)	0.482
Pulmonary bacterial infection	6 (16.7%)	31 (27.0%)	0.210	6 (17.1%)	23 (26.4%)	0.275
Pulmonary fungal infection	0 (0.0%)	5 (4.3%)	0.339	0 (0.0%)	3 (3.4%)	0.557
Carcinoma	5 (13.9%)	19 (16.5%)	0.706	5 (14.3%)	14 (16.1%)	0.803
UIP pattern	9 (25.0%)	26 (22.6%)	0.767	8 (22.9%)	20 (23.0%)	0.988
Pneumomediastinum	1 (2.8%)	6 (5.2%)	0.878	1 (2.9%)	4 (4.6%)	1.000
Survival^a^ (m)	4 (11.1%)	38 (33.0%)	0.015	4 (14.4%)	30 (34.5%)	0.016
RP-ILD	2 (5.6%)	27 (23.5%)	0.017	2 (5.7%)	22 (25.3%)	0.014
**Disease activity**						
MYOACT score	8.0 (7.0, 9.0)	8.0 (6.0, 10.0)	0.635	8.0 (7.0, 9.0)	8.0 (6.0, 10.0)	0.649
**Lung function testing**						
FVC% (%)	65.1 ± 16.4	70.8 ± 18.6	0.103	64.7 ± 16.4	70.4 ± 18.5	0.110
TLC (L)	3.6 ± 1.2	3.7 ± 1.0	0.465	3.5 ± 1.2	3.7 ± 1.0	0.535
FEV1% (%)	68.5 ± 16.5	72.3 ± 19.4	0.286	68.0 ± 16.6	71.4 ± 19.4	0.370
FEV1/FVC	0.8 (0.8, 0.9)	0.8 (0.8, 0.9)	0.218	0.8 (0.8, 0.9)	0.8 (0.8, 0.9)	0.105
DLCO% (%)	53.0 ± 20.8	60.6 ± 19.7	0.049	53.0 ± 21.1	58.1 ± 18.1	0.184
**Myositis-specific antibodies and myositis-associated antibodies**						
Anti-MDA5	9 (25.0%)	25 (21.7%)	0.683	9 (25.7%)	21 (24.1%)	0.855
Anti-PL-7	3 (8.3%)	16 (13.9%)	0.553	2 (5.7%)	13 (14.9%)	0.272
Anti-PL-12	1 (2.8%)	4 (3.5%)	1.000	1 (2.9%)	3 (3.4%)	1.000
Anti-EJ	3 (8.3%)	6 (5.2%)	0.775	3 (8.6%)	4 (4.6%)	0.408
Anti-Ro-52	21 (58.3%)	59 (51.3%)	0.461	21 (60.0%)	47 (54.0%)	0.548
Anti-Jo-1	5 (13.9%)	21 (18.3%)	0.544	5 (14.3%)	19 (21.8%)	0.342
Anti-OJ	2 (5.6%)	2 (1.7%)	0.241	2 (5.7%)	2 (2.3%)	0.578
Anti-TIF1γ	1 (2.8%)	13 (11.3%)	0.226	1 (2.9%)	11 (12.6%)	0.192
Anti-Ku	1 (2.8%)	6 (5.2%)	0.878	1 (2.9%)	4 (4.6%)	1.000
Anti-SRP	2 (5.6%)	4 (3.5%)	0.629	2 (5.7%)	3 (3.4%)	0.624
Anti-NXP2	1 (2.8%)	11 (9.6%)	0.337	1 (2.9%)	7 (8.0%)	0.520
Anti-PM-Scl75	3 (8.3%)	9 (7.8%)	1.000	3 (8.6%)	6 (6.9%)	1.000
Anti-PM-Scl100	1 (2.8%)	2 (1.7%)	0.561	0 (0.0%)	2 (2.3%)	1.000
Anti-Mi-2α	3 (8.3%)	6 (5.2%)	0.775	3 (8.6%)	2 (2.3%)	0.142
Anti-Mi-2β	1 (2.8%)	7 (6.1%)	0.728	1 (2.9%)	3 (3.4%)	1.000
Anti-SAE1	2 (5.6%)	4 (3.5%)	0.629	2 (5.7%)	3 (3.4%)	0.624
**Comorbidities/harmful hobbies**						
Smoking	7 (19.4%)	15 (13.0%)	0.342	7 (20.0%)	11 (12.6%)	0.300
Alcohol abuse	7 (19.4%)	23 (20.0%)	0.942	7 (20.0%)	16 (18.4%)	0.837
Hypertension	10 (27.8%)	15 (13.0%)	0.038	9 (25.7%)	14 (16.1%)	0.219
Diabetes	8 (22.2%)	18 (15.7%)	0.362	8 (22.9%)	15 (17.2%)	0.473
Hepatitis	6 (16.7%)	19 (16.5%)	0.984	6 (17.1%)	16 (18.4%)	0.871
Allergic history	5 (13.9%)	18 (15.7%)	0.797	5 (14.3%)	9 (10.3%)	0.761
**Therapies**						
Steroid monotherapy	7 (19.4%)	28 (24.3%)	0.543	7 (20.0%)	21 (24.1%)	0.623
Steroid + DMARDs	23 (63.9%)	57 (49.6%)	0.133	22 (62.9%)	44 (50.6%)	0.218
Steroid + IVIG	3 (8.3%)	17 (14.8%)	0.475	3 (8.6%)	13 (14.9%)	0.518
Steroid + DMARDs + IVIG	3 (8.3%)	13 (11.3%)	0.845	3 (8.6%)	9 (10.3%)	1.000
Maximum dosage of steroid^b^	50.0 (38.8, 100.0)	75.0 (50.0, 100.0)	0.112	50.0 (35.0, 100.0)	75.0 (50.0, 100.0)	0.139
**IIM subtypes**						
DM	24 (66.7%)	72 (62.6%)	0.659	23 (65.7%)	54 (62.1%)	0.706
PM	7 (19.4%)	29 (25.2%)	0.478	7 (20.0%)	21 (24.1%)	0.623
ADM	5 (13.9%)	14 (12.2%)	1.000	5 (14.3%)	12 (13.8%)	1.000

*y, years; m, months; UIP pattern, Usual interstitial pneumonia pattern; RP-ILD, Rapidly progressive interstitial lung disease; MYOACT, Myositis Disease Activity Assessment Visual Analog Scales; FVC%, Percent-predicted forced vital capacity; TLC, Total lung capacity; L, Liter; FEV1%, Percent-predicted forced expiratory volume in 1 s; FEV1/FVC, Ratio of FEV1 over FVC; DLCO%, Percent-predicted diffusing capacity of the lung for carbon monoxide; DMARDs, Disease-modifying anti-rheumatic drugs; IVIG, Intravenous immunoglobulin; IIM, Idiopathic inflammatory myopathy; DM, dermatomyositis; PM, Polymyositis; ADM, Amyopathic dermatomyositis*.

a *Mortality in follow-up, P-value was calculated using Kaplan-Meier method with log-rank test*.

b *Calculated by prednisolone*.

To further evaluate the effect of nintedanib in risk of RP-ILD, univariate and multivariate logistic regression analyses were utilized to adjust for other clinical factors. In univariate analysis, MYOACT score (*P* < 0.001), DLCO% (*P* = 0.003), positivity of anti-MDA5 antibody (*P* = 0.009), use of steroid and IVIG (*P* = 0.015), nintedanib therapy (*P* = 0.030, OR value = 0.192) as well as ADM (*P* = 0.010) were found to be significantly correlated with risk of RP-ILD. The following multivariate logistic regression analysis revealed that MYOACT score (*P* < 0.001), DLCO% (*P* = 0.036), nintedanib therapy (*P* = 0.004, OR value = 0.072) and ADM (*P* = 0.012) were significantly correlated with risk of RP-ILD in patients with IIM-ILD. Similar results were identified after propensity score matching. MYOACT score (*P* < 0.001), nintedanib therapy (*P* = 0.009, OR value = 0.093) and ADM (*P* = 0.003) were still significantly associated with RP-ILD in these patients (Table 2).

**Table 2:** Univariate and multivariate logistic regression analysis of RP-ILD in IIM-ILD patients.

**Factors**	**Before propensity score matching**			**After propensity score matching**		
	***P*-value**	**OR value**	**95%Cl**	***P*-value**	**OR value**	**95% Cl**
**Univariate analysis**						
Age (y)	0.626	1.009	0.973~1.046	0.248	1.024	0.983~1.067
Sex (male/female)	0.851	1.083	0.469~2.500	0.525	1.345	0.540~3.347
**Clinical manifestations or comorbidities**						
Dysphagia	0.420	0.588	0.162~2.133	0.514	0.594	0.125~2.831
Dysarthria	0.456	0.448	0.055~3.688	0.999	<0.001	0.000~
Respiratory muscle involvement	0.963	1.054	0.113~9.795	0.999	<0.001	0.000~
Cardiac involvement	0.625	0.587	0.069~4.965	0.714	0.667	0.076~5.814
Gastrointestinal hemorrhage	0.999	<0.001	0.000~	0.999	<0.001	0.000~
Pulmonary bacterial infection	0.168	1.852	0.770~4.451	0.875	1.087	0.386~3.061
Pulmonary fungal infection	0.963	1.054	0.113~9.795	0.555	2.087	0.181~24.020
Carcinoma	0.825	1.129	0.383~3.328	0.431	1.579	0.507~4.918
UIP pattern	0.724	1.195	0.444~3.218	0.790	0.868	0.307~2.454
Pneumomediastinum	0.123	3.404	0.718~16.134	0.985	1.022	0.109~9.581
**Disease activity**						
MYOACT score	<0.001	1.336	1.167~1.530	<0.001	1.316	1.138~1.522
**Lung function testing**						
FVC% (%)	0.600	0.994	0.972~1.017	0.766	1.004	0.979~1.029
TLC (L)	0.057	0.668	0.442~1.012	0.157	0.720	0.457~1.135
FEV1% (%)	0.074	0.979	0.957~1.002	0.315	0.987	0.963~1.012
FEV1/FVC	0.240	0.086	0.001~5.171	0.123	0.014	0.000~3.162
DLCO% (%)	0.003	0.964	0.941~0.987	0.010	0.964	0.937~0.991
**Myositis-specific antibodies and myositis-associated antibodies**						
Anti-MDA5	0.009	3.209	1.343~7.668	0.009	3.518	1.366~9.064
Anti-PL-7	0.687	0.764	0.207~2.821	0.514	0.594	0.125~2.831
Anti-PL-12	0.250	2.938	0.468~18.452	0.152	4.364	0.582~32.697
Anti-EJ	0.278	2.231	0.523~9.507	0.546	1.691	0.308~9.297
Anti-Ro-52	0.499	1.327	0.584~3.012	0.775	1.141	0.462~2.817
Anti-Jo-1	0.588	0.727	0.230~2.301	0.331	0.524	0.142~1.927
Anti-OJ	0.999	<0.001	0.000~	0.999	<0.001	0.000~
Anti-TIF1γ	0.626	0.679	0.143~3.215	0.783	0.800	0.163~3.917
Anti-Ku	0.736	0.690	0.080~5.968	0.985	1.022	0.109~9.581
Anti-SRP	0.999	<0.001	0.000~	0.999	<0.001	0.000~
Anti-NXP2	0.999	<0.001	0.000~	0.999	<0.001	0.000~
Anti-PM-Scl75	0.205	2.280	0.637~8.167	0.066	3.720	0.917~15.096
Anti-PM-Scl100	0.999	<0.001	0.000~	0.999	<0.001	0.000~
Anti-Mi-2α	0.532	0.509	0.061~4.238	0.999	<0.001	0.000~
Anti-Mi-2β	0.670	1.432	0.274~7.488	0.786	1.377	0.137~13.850
Anti-SAE1	0.999	<0.001	0.000~	0.999	<0.001	0.000~
**Comorbidities/harmful hobbies**						
Smoking	0.895	0.924	0.287~2.973	0.768	1.200	0.357~4.038
Alcohol abuse	0.694	0.808	0.280~2.331	0.782	1.170	0.385~3.549
Hypertension	0.657	0.770	0.242~2.444	0.760	0.832	0.254~2.719
Diabetes	0.583	1.330	0.481~3.683	0.393	1.588	0.549~4.592
Hepatitis	0.507	1.414	0.508~3.934	0.691	1.254	0.411~3.825
Allergic History	0.181	0.356	0.079~1.615	0.999	<0.001	0.000~
**Therapies**						
Steroid monotherapy	0.411	1.001	0.999~1.004	0.555	1.001	0.998~1.003
Steroid + DMARDs	0.167	0.561	0.247~1.274	0.026	0.345	0.135~0.882
Steroid + IVIG	0.015	3.492	1.273~9.579	0.002	5.625	1.845~17.153
Steroid + DMARDs + IVIG	0.961	0.967	0.257~3.644	0.783	0.800	0.163~3.917
Maximum dosage of steroid^a^	0.363	1.001	0.998~1.005	0.527	1.001	0.998~1.004
Nintedanib	0.030	0.192	0.043~0.851	0.025	0.179	0.040~0.808
**IIM subtypes**						
DM	0.143	0.542	0.239~1.231	0.313	0.628	0.254~1.551
PM	0.658	0.800	0.298~2.150	0.185	0.417	0.115~1.519
ADM	0.010	3.844	1.382~10.695	0.004	4.944	1.661~14.721
**Multivariate analysis**						
MYOACT score	<0.001	1.346	1.151~1.574	<0.001	1.392	1.175~1.650
DLCO%	0.036	0.970	0.943~0.998			
Nintedanib	0.004	0.072	0.012~0.426	0.009	0.093	0.016~0.549
ADM	0.012	5.192	1.433~18.808	0.003	7.343	2.007~26.865

*RP-ILD, Rapidly progressive interstitial lung disease; IIM-ILD, Idiopathic-inflammatory-myopathy-related interstitial lung disease; y, years; m, months; UIP pattern, Usual interstitial pneumonia pattern; MYOACT, Myositis Disease Activity Assessment Visual Analog Scales; FVC%, Percent-predicted forced vital capacity; TLC, Total lung capacity; L, Liter; FEV1%, Percent-predicted forced expiratory volume in 1 s; FEV1/FVC, Ratio of FEV1 over FVC; DLCO%, Percent-predicted diffusing capacity of the lung for carbon monoxide; DMARDs, Disease-modifying anti-rheumatic drugs; IVIG, Intravenous immunoglobulin; IIM, Idiopathic inflammatory myopathy; DM, dermatomyositis; PM, Polymyositis; ADM, Amyopathic dermatomyositis*.

a *Calculated by prednisolone*.

In the setting of time to death from any cause, the univariate Cox proportional hazards regression analysis identified pulmonary bacterial infection (*P* = 0.005), RP-ILD (*P* < 0.001), MYOACT score (*P* < 0.001), DLCO% (*P* = 0.006), positivity of anti-MDA5 antibody (*P* < 0.001) and nintedanib therapy (*P* = 0.023) as factors significantly related to survival in follow-up. The subsequent multivariate analysis showed that nintedanib therapy improved the survival of IIM-ILD patients (*P* = 0.013 HR value = 0.268) after adjusting for other clinical factors. Besides, elevated MYOACT score (*P* < 0.001) and positivity of anti-MDA5 antibody (*P* < 0.001) were found to be risk factors for death in IIM-ILD patients. After propensity score matching, MYOACT score (*P* = 0.001), positivity of anti-MDA5 antibody (*P* < 0.001) and nintedanib therapy (*P* = 0.013, HR value = 0.264) remain statistically significant factors for death in these patients (Table 3).

**Table 3:** Univariate and multivariate Cox proportional hazards regression analysis of survival in IIM-ILD patients.

	**Before propensity score matching**			**After propensity score matching**		
**Factors**	***P*-value**	**HR value**	**95% Cl**	***P*-value**	**HR value**	**95% Cl**
Age (y)	0.111	1.023	0.995~1.052	0.059	1.031	0.999~1.064
Sex (male/female)	0.872	0.950	0.505~1.785	0.819	0.921	0.455~1.863
**Clinical manifestations or comorbidities**						
Dysphagia	0.091	1.846	0.907~3.756	0.054	2.278	0.987~5.257
Dysarthria	0.613	1.305	0.465~3.666	0.504	0.507	0.069~3.714
Respiratory muscle involvement	0.240	2.022	0.624~6.554	0.610	1.679	0.229~12.294
Cardiac involvement	0.166	2.076	0.739~5.830	0.077	2.576	0.903~7.346
Gastrointestinal hemorrhage	0.165	1.940	0.760~4.950	0.395	1.575	0.553~4.489
Pulmonary bacterial infection	0.005	2.435	1.311~4.525	0.025	2.225	1.107~4.473
Pulmonary fungal infection	0.674	1.357	0.328~5.626	0.190	2.601	0.622~10.878
Carcinoma	0.868	1.071	0.476~2.412	0.837	0.905	0.350~2.338
UIP pattern	0.239	1.629	0.723~3.671	0.417	1.441	0.596~3.483
Pneumomediastinum	0.868	1.128	0.272~4.675	0.385	0.046	0.000~47.796
RP-ILD	<0.001	3.930	2.108~7.327	<0.001	3.927	1.964~7.851
**Disease activity**						
MYOACT score	<0.001	1.261	1.156~1.375	<0.001	1.226	1.114~1.348
**Lung function testing**						
FVC% (%)	0.228	0.990	0.973~1.006	0.561	0.995	0.976~1.013
TLC (L)	0.103	0.773	0.568~1.054	0.346	0.850	0.606~1.192
FEV1% (%)	0.134	0.988	0.972~1.004	0.327	0.991	0.973~1.009
FEV1/FVC	0.814	1.415	0.079~25.465	0.882	0.740	0.014~39.720
DLCO% (%)	0.006	0.977	0.960~0.993	0.042	0.979	0.960~0.999
**Myositis-specific antibodies and myositis-associated antibodies**						
Anti-MDA5	<0.001	4.318	2.324~8.023	<0.001	4.734	2.372~9.451
Anti-PL-7	0.481	0.690	0.246~1.935	0.518	0.676	0.206~2.217
Anti-PL-12	0.696	0,673	0.093~4.902	0.917	0.899	0.123~6.586
Anti-EJ	0.718	1.242	0.383~4.026	0.985	1.014	0.242~4.240
Anti-Ro-52	0.374	1.320	0.716~2.436	0.326	1.415	0.707~2.831
Anti-Jo-1	0.455	0.719	0.303~1.709	0.575	0.777	0.321~1.879
Anti-OJ	0.862	1.192	0.163~8.696	0.890	1.152	0.157~8.453
Anti-TIF1γ	0.813	0.883	0.315~2.479	0.644	0.756	0.230~2.479
Anti-Ku	0.977	1.021	0.246~4.229	0.547	1.553	0.371~6.494
Anti-SRP	0.357	0.046	0.000~31.881	0.406	0.046	0.000~64.547
Anti-NXP2	0.167	0.247	0.034~1.794	0.342	0.381	0.052~2.791
Anti-PM-Scl75	0.817	1.130	0.402~3.175	0.331	1.681	0.590~4.791
Anti-PM-Scl100	0.838	1.234	0.166~9.179	0.257	3.175	0.431~23.391
Anti-Mi-2α	0.748	0.792	0.191~3.281	0.404	0.046	0.000~62.827
Anti-Mi-2β	0.942	0.949	0.229~3.933	0.467	0.047	0.000~178.915
Anti-SAE1	0.551	0.546	0.075~3.988	0.637	0.619	0.084~4.556
**Comorbidities/harmful hobbies**						
Smoking	0.717	0.841	0.330~2.145	0.716	0.824	0.289~2.344
Alcohol abuse	0.867	1.065	0.508~2.232	0.778	1.127	0.489~2.598
Hypertension	0.576	0.781	0.329~1.855	0.791	0.888	0.368~2.144
Diabetes	0.540	0.763	0.321~1.812	0.457	0.697	0.269~1.804
Hepatitis	0.816	1.096	0.507~2.370	0.830	0.908	0.375~2.197
Allergic history	0.053	0.246	0.059~1.017	0.143	0.040	0.001~2.968
**Therapies**						
Steroid monotherapy	0.065	1.001	1.000~1.003	0.141	1.001	1.000~1.002
Steroid + DMARDs	0.623	0.859	0.469~1.574	0.163	0.618	0.314~1.216
Steroid + IVIG	0.390	1.429	0.633~3.225	0.088	2.070	0.898~4.775
Steroid + DMARDs + IVIG	0.288	1.600	0.672~3.808	0.166	1.962	0.755~5.094
Maximum dosage of steroid	0.059	1.002	1.000~1.003	0.134	1.001	1.000~1.003
Nintedanib	0.023	0.301	0.107~0.845	0.023	0.299	0.105~0.849
**IIM subtypes**						
DM	0.744	0.901	0.483~1.681	0.503	0.792	0.400~1.568
PM	0.638	0.838	0.401~1.752	0.662	0.830	0.361~1.910
ADM	0.257	1.602	0.709~3.617	0.122	1.934	0.838~4.465
**Multivariate analysis**						
MYOACT score	<0.001	1.214	1.110~1.328	0.001	1.174	1.068~1.291
Anti-MDA5	<0.001	3.300	1.714~6.357	<0.001	4.170	2.011~8.649
Nintedanib	0.013	0.268	0.096~0.754	0.013	0.264	0.093~0.753

*IIM-ILD, Idiopathic-inflammatory-myopathy-related interstitial lung disease; y, years; m, months; UIP pattern, Usual interstitial pneumonia pattern; RP-ILD, Rapidly progressive interstitial lung disease; MYOACT, Myositis Disease Activity Assessment Visual Analog Scales; FVC%, Percent-predicted forced vital capacity; TLC, Total lung capacity; L, Liter; FEV1%, Percent-predicted forced expiratory volume in 1 s; FEV1/FVC, Ratio of FEV1 over FVC; DLCO%, Percent-predicted diffusing capacity of the lung for carbon monoxide; DMARDs, Disease-modifying anti-rheumatic drugs; IVIG, Intravenous immunoglobulin; IIM, Idiopathic inflammatory myopathy; DM, dermatomyositis; PM, Polymyositis; ADM, Amyopathic dermatomyositis*.

*^a^Calculated by prednisolone*.

**Figure 3:**
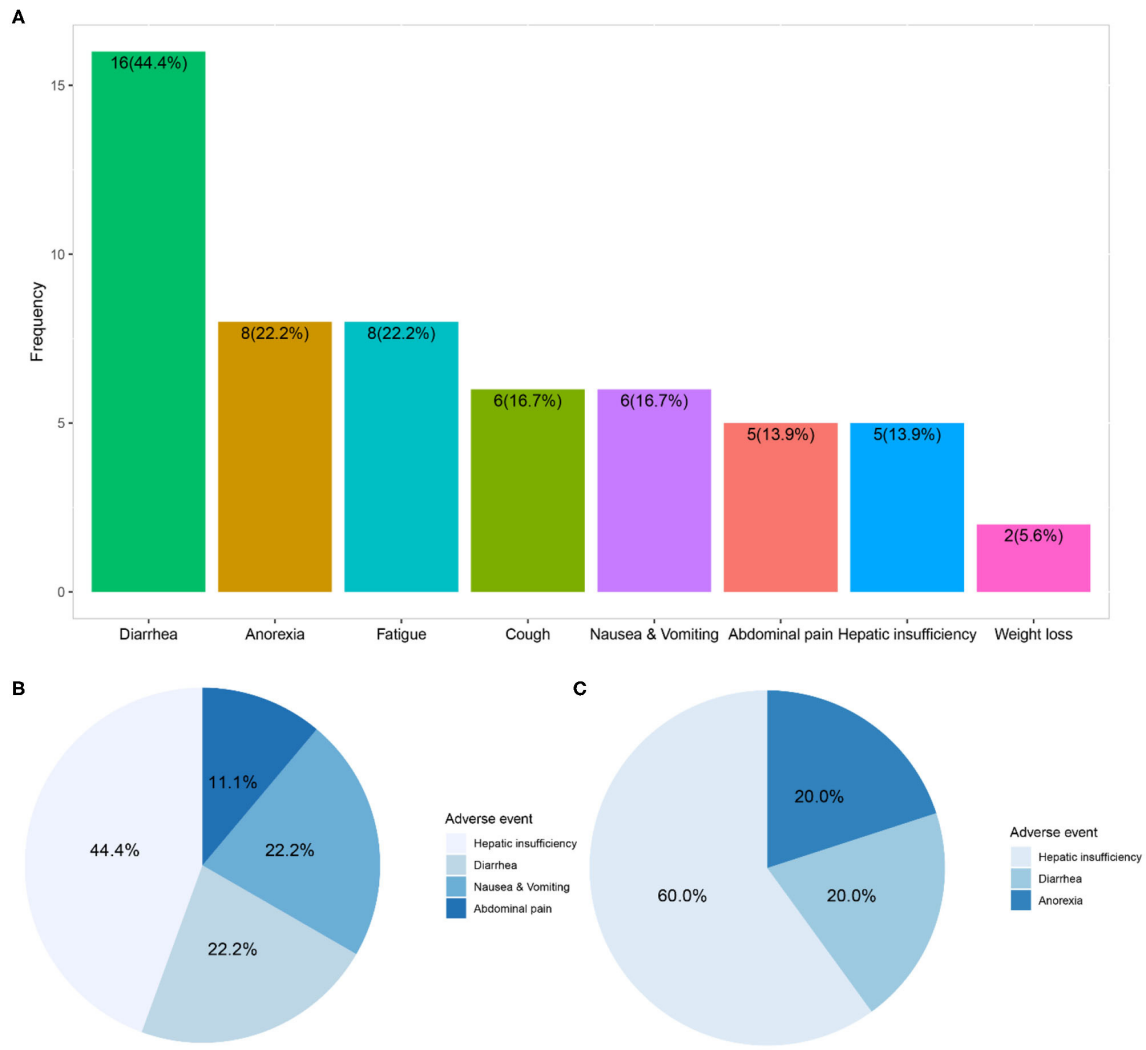
Adverse events and direct contribution to dosage reduction or therapy discontinuation of nintedanib. **(A)** Showed distribution of adverse events within 36 patients receiving compassionate nintedanib therapy; **(B)** revealed distribution of adverse events directly related to dosage reduction of nintedanib in 9 patients; **(C)** exhibited distribution of adverse events directly related to therapy discontinuation of nintedanib in 5 patients.

In the 36 patients receiving nintedanib therapy, nintedanib was initially administered 150 mg twice daily as per the manufacturer's recommendation. The dosage was reduced to 100 mg twice daily due to adverse events in 9 patients. And 9 patients finally terminated nintedanib therapy (5 due to intolerable adverse events and 4 due to mortality). Dosage reduction or therapy discontinuation occurred to 44.4% of nintedanib-treated patients. During a medium nintedanib therapy period of 11.0 (6.3, 20.2) months, adverse events occurred to 69.4% of patients, and diarrhea was the most common adverse event (44.4%), followed by anorexia (22.2%) and fatigue (22.2%). Although hepatic insufficiency only happened to 5 patients (13.9%), it was the major adverse event directly associated with dosage reduction (44.4% of nine patients) or therapy discontinuation (60.0% of five patients) in the follow-up interviews (Figure 3 and Supplementary Table 2).

## Discussion

Nintedanib is an intracellular inhibitor that targets multiple tyrosine kinases and hereby inhibits fundamental processes in the pathogenesis of fibrosis. Although proven to be effective in IPF, Ssc-related ILD and other autoimmune ILD, the efficacy and tolerability of nintedanib in the subtype of IIM-ILD has not been clarified. In this study, all IIM-ILD patients received potent immunosuppressive therapy. However, the incidence of RP-ILD still amounted to 23.5% in the control group, which is similar to the incidence of rapid progression/acute exacerbation in previous reports of IIM-ILD (24–38%) and IPF (8.6–23.9%), but higher than the incidence in Ssc-ILD (9.4%) (10–13). Efficacy and tolerability of nintedanib in IIM-ILD patients were hereby a worthy subject. To date, this is the first clinical research focusing on nintedanib therapy in IIM-ILD patients. Nintedanib was found effective in reducing incidence of RP-ILD and improving survival in these patients. However, patients receiving nintedanib therapy did not suffer from less comorbidity of pulmonary infection. Tolerability of nintedanib therapy was as well-evaluated by recording and discussing the adverse events, dosage reduction and discontinuation of the therapy. Admittedly the non-randomized process of inclusion contributed to the heterogeneity between the two groups. Patients who accepted nintedanib therapy tended to have higher disease activity (as measured by MYOACT score) as well as worse lung involvement (as measured by DLCO%). However, propensity score matching was used to adjust for the heterogeneity between the two groups. After propensity score matching, less RP-ILD and better survival could still be identified in patients receiving nintedanib therapy.

Nintedanib has already been proved of therapeutic value in reducing lung function decline and acute exacerbation of idiopathic pulmonary fibrosis (AE-IPF) in IPF patients. The previous TOMORROW trial, INPULSIS-1 and INPULSIS-2 trials demonstrated that nintedanib promisingly reduced the annual rate of decline in FVC (21, 22). The therapeutic effect of nintedanib remain consistent irrespective of age, sex, ethnicity, and baseline percent-predicted forced vital capacity (FVC%) (35, 36), and the efficacy in slowing lung function decline could last over 3 years (37). Similar results were also reported in several real-world experience across Europe during the Compassionate Use Programs (CUPs) or after license (38–40). In addition, the TOMORROW trial and INPULSIS-2 trial also demonstrated that nintedanib played a protective role in preventing AE-IPF (41). In INPULSIS-2 trial, nintedanib significantly increased the time to the first acute exacerbation. Meanwhile the incidence of acute exacerbation was reduced in TOMORROW trial. The following INPULSIS-ON trial also suggested that nintedanib's therapeutic effect in reducing occurrence of acute exacerbation persisted beyond 3 years. In SENSCIS trial, nintedanib was identified to decrease FVC decline in Ssc-ILD, nevertheless, its effect on RP-ILD was not probed into (24). The recently completed INBUILD trial demonstrated the consistent effect of nintedanib on reducing FVC decline in different ILD subgroups, irrespective of the underlying ILD diagnosis (26). In our study, nintedanib was found to reduce the incidence of the fatal RP-ILD in IIM-ILD patients, which for the first time suggested the therapeutic value of nintedanib in IIM-ILD regardless of the retrospective nature and lack of follow-up lung function testing. In the future, large-cohort, prospective, multicenter research concerning IIM-ILD is demanded to verify the role of nintedanib in decreasing incidence of RP-ILD, as well as explore its effect in lung function decline, etc.

In addition to decreasing the incidence of RP-ILD in follow-up, nintedanib was also observed to benefit the survival of IIM-ILD patients. Similar finding in IPF patients was also reported in an Italian multicenter study with a favorable 1-year survival of 79% (42). On the one hand, RP-ILD has been identified as a risk factor for death in IIM-ILD patients (14, 15). Nintedanib might improve IIM patients' survival by reducing the incidence of RP-ILD. On the other hand, nintedanib was still found to play a protective role in survival after adjusting for RP-ILD, etc., which led us to consider the possible therapeutic value of nintedanib beyond ILD. In preclinical models of systemic sclerosis, nintedanib was confirmed to alleviate skin fibrosis by decreasing of dermal thickness, myofibroblast counts and hydroxyproline content (43). However, no clinical benefit of nintedanib was identified for extra-pulmonary manifestations of systemic sclerosis in SENSCIS trial (24). In terms of IIM, preceding studies proposed the essential role of tyrosine kinase in type I IFN signaling in pathogenesis of DM (44). As a tyrosine kinase inhibitor (45), the effect of nintedanib on disease activity, muscular or extra-muscular involvements as well as pathogenesis of IIM, DM in particular, is worth exploration in the future. The benefit of nintedanib in survival of IIM-ILD patients demanded further verification in longer therapeutic regimen and follow-up period.

During the course of nintedanib therapy, adverse events were inevitable in most patients and might impede the therapeutic regimen by dosage reduction or discontinuation of therapy. In our study, 69.4% of IIM-ILD patients receiving nintedanib therapy suffered from adverse events. Almost half of nintedanib-treated patients finally received reduced dosage of nintedanib or discontinued the therapy. The incidence of adverse events was much lower than those (~90%) reported in preceding clinical trials possibly owing to exclusion of ILD progression, upper respiratory tract infection, etc. from adverse events (21, 22, 24, 26). Diarrhea was the most common adverse event in IIM-ILD patients, which resembled the reports from series of clinical trials and real-world analyses (21–23, 26, 38–40, 46). Besides, liver function monitoring and timely liver-protected medication should be emphasized in IIM-ILD patients since hepatic insufficiency was identified as the major direct contributor to dosage reduction or discontinuation due to adverse events.

MSAs and MAAs are two main subtypes of antibodies in IIM patients. They were valuable in the field of diagnosis and evaluation. In preceding studies, anti-MDA5 antibody was found to be correlated with ILD, RP-ILD as well as death in DM or ADM patients (47–49). In this study, anti-MDA5 antibody was recognized as a risk factor for death in IIM-ILD patients. Anti-MDA5 antibody was as well found to be significantly correlated with RP-ILD in univariate analysis. However, the significance vanished after adjusting for disease activity, etc., possibly owing to the relatively high disease activity of patients with positivity of anti-MDA5 antibody.

Besides, lower DLCO% and ADM were found to be correlated with occurrence of RP-ILD. Lower DLCO% seemed to be both initiating factor and consequence of progression of ILD. On the one hand, lower DLCO% indicated decreased gas-exchanging function of lung and the subsequent hypoxia. Hypoxia might induce progression of ILD via augmenting oxidative and inflammatory pathways, increasing the total lung collagen content and heterogeneous structural alterations (50, 51). On the other hand, lower DLCO% could be taken as an early-stage manifestation of progression of ILD since ILD and its progression could result in impaired diffuse capacity through alveolar structural alteration, thickening of alveolar capillary wall, etc. However, the unsatisfying OR value of DLCO% in predicting RP-ILD made it less valuable in clinical practice. Previous studies also proposed that RP-ILD was more frequently seen in ADM patients, possibly owing to the higher CD4/CD8 ratio in ADM-related ILD patients (48–50). ILD patients with higher proportion of CD4+ T cell in BALF and peripheral blood tended to be rapidly progressive (52, 53). Similar elevated ratio can also be seen in BALF of ADM-related ILD patients (54). Confirmation of the role and mechanism of higher CD4/CD8 ratio in RP-ILD demanded further exploration.

Elevated MYOACT score, which reflected higher disease activity in IIM patients, was found to be correlated with RP-ILD and death. As a core set measure for IIM patients, MYOACT score work as an overall assessment of IIM disease activity by taking muscular and various extra-muscular manifestations into consideration. It has been suggested to predict outcome of IIM patients and reflect inflammatory level (55, 56). Meanwhile RP-ILD was also found to be associated with cytokines like tumor necrosis factor, etc. (57). The partially overlapped pathological mechanism might make MYOACT score a satisfying predictor for RP-ILD in IIM-ILD patients.

The most significant limitations of this study were the retrospective and observational nature as well as the small sample size. Due to the initial lack of follow-up lung function testing and recent impact of COVID-19 pandemic, we failed to clarify the effect of nintedanib on lung function decline. The judgment of RP-ILD mostly relied on progression of symptoms and HRCT alterations, instead of worsening of lung function testing. Alterations of life quality, MYOACT score, muscle strength, etc. after nintedanib therapy were not recorded as well. Baseline FVC%, DLCO%, age, immunosuppressive therapy, etc. were not controlled for inclusion because of the limited number of IIM-ILD patients receiving nintedanib therapy. No placebo was given to patients in the control group. Last but not least, underestimation of RP-ILD might occur in patients under conventional medications owing to initial lack of mandatory management. In spite of all the limitations, we intended to initially figure out the therapeutic value of nintedanib in IIM-ILD, and shed some light on the future therapeutic landscape of CTD-ILD.

## Conclusions

In IIM-ILD patients, nintedanib might be effective in reducing incidence of RP-ILD as well as improving survival. The efficacy of nintedanib in lung function decline, life quality and disease activity of IIM, etc. demands exploration in future study. Besides, anti-MDA5 antibody was verified to be a risk factor for death in IIM-ILD patients, and ADM might be valuable in predicting occurrence of RP-ILD. Among IIM-ILD patients receiving nintedanib therapy, diarrhea was the most common adverse event. Hepatic insufficiency frequently led to dosage reduction or therapy discontinuation.

## Data Availability Statement

The original contributions presented in the study are included in the article/Supplementary Material, further inquiries can be directed to the corresponding author.

## Ethics Statement

Written informed consent was obtained from the individual(s) for the publication of any potentially identifiable images or data included in this article.

## Author Contributions

JLia, HC, and JLin contributed to the design of the work. JLia, HC, YK, CS, LY, YYa, and YYu performed the data acquisition. JLia and HC performed the data analysis and contributed to the interpretation of the data and drafting the work. All authors revised the work critically for important intellectual content and all finally approved the version to be submitted and published.

## Conflict of Interest

The authors declare that the research was conducted in the absence of any commercial or financial relationships that could be construed as a potential conflict of interest.
