# Supplementary material for: Efficacy and Tolerability of Nintedanib in Idiopathic-Inflammatory-Myopathy-Related Interstitial Lung Disease: A Pilot Study
Source: Front Med (Lausanne). 2021 Feb 3;8:626953. doi: 10.3389/fmed.2021.626953 (PMC7886679; doi:10.3389/fmed.2021.626953)
Supplement: Supplementary file 1 [file Table_1.DOCX]

**Supplementary table 1: Brief overview of 36 IIM-ILD patients receiving compassionate nintedanib therapy.**

IIM-ILD: Idiopathic-inflammatory-myopathy-related interstitial lung disease; y: years; m: months; RP-ILD: Rapidly progressive interstitial lung disease; Y: Yes; N: No.

| **Coding** | **Age (y)** | **Sex** | **Duration of nintedanib therapy (m)** | **Adverse event** | **Diarrhea** | **Abdominal pain** | **Nausea & Vomiting** | **Anorexia** | **Weight loss** | **Fatigue** | **Hepatic insufficiency** | **Cough** | **Dosage reduction** | **Direct reason for dosage reduction** | **Therapy discontinuation** | **Direct reason for therapy discontinuation** | **Death from any cause** | **Follow-up (m)** | **RP-ILD** |
| --- | --- | --- | --- | --- | --- | --- | --- | --- | --- | --- | --- | --- | --- | --- | --- | --- | --- | --- | --- |
| **1** | **33** | **Female** | **12.2** | **Y** | **●** |  |  |  |  |  | **●** |  | **Y** | **Hepatic insufficiency** | **Y** | **Hepatic insufficiency** | **N** | **30.16** | **N** |
| **2** | **70** | **Female** | **5.5** | **Y** | **●** |  | **●** | **●** | **●** | **●** | **●** |  | **Y** | **Hepatic insufficiency** | **Y** | **Hepatic insufficiency** | **N** | **14.43** | **N** |
| **3** | **64** | **Male** | **24.6** | **Y** | **●** |  |  |  |  |  |  | **●** | **Y** | **Diarrhea** | **N** |  | **N** | **24.6** | **N** |
| **4** | **62** | **Female** | **29.0** | **Y** | **●** | **●** |  |  |  | **●** |  |  | **Y** | **Abdominal pain** | **N** |  | **N** | **29.0** | **N** |
| **5** | **57** | **Female** | **13.1** | **Y** |  |  |  | **●** |  |  | **●** |  | **Y** | **Hepatic insufficiency** | **N** |  | **N** | **13.1** | **N** |
| **6** | **61** | **Male** | **24.6** | **Y** | **●** |  |  |  |  |  |  |  | **Y** | **Diarrhea** | **N** |  | **N** | **24.6** | **N** |
| **7** | **42** | **Male** | **0.93** | **Y** | **●** |  |  |  |  | **●** |  |  | **N** |  | **Y** | **Death** | **Y** | **0.93** | **Y** |
| **8** | **41** | **Female** | **6.5** | **Y** | **●** | **●** |  | **●** |  |  |  |  | **N** |  | **Y** | **Anorexia** | **N** | **13.1** | **N** |
| **9** | **61** | **Male** | **7.5** | **Y** | **●** |  |  |  |  |  |  |  | **N** |  | **Y** | **Diarrhea** | **N** | **8.83** | **N** |
| **10** | **56** | **Female** | **1.5** | **N** |  |  |  |  |  |  |  |  | **N** |  | **Y** | **Death** | **Y** | **1.5** | **N** |
| **11** | **53** | **Male** | **18.83** | **Y** | **●** |  |  |  |  | **●** |  | **●** | **N** |  | **N** |  | **N** | **18.83** | **N** |
| **12** | **53** | **Female** | **20.7** | **Y** |  |  |  |  |  |  |  | **●** | **N** |  | **N** |  | **N** | **20.7** | **N** |
| **13** | **65** | **Male** | **28.3** | **Y** | **●** |  |  |  |  |  |  | **●** | **N** |  | **N** |  | **N** | **28.3** | **N** |
| **14** | **41** | **Male** | **13.6** | **Y** | **●** |  |  |  |  |  |  |  | **N** |  | **N** |  | **N** | **13.6** | **N** |
| **15** | **41** | **Male** | **11.7** | **Y** | **●** | **●** |  |  |  |  |  |  | **N** |  | **N** |  | **N** | **11.7** | **N** |
| **16** | **65** | **Female** | **13.6** | **N** |  |  |  |  |  |  |  |  | **N** |  | **N** |  | **N** | **13.6** | **N** |
| **17** | **50** | **Female** | **24.57** | **N** |  |  |  |  |  |  |  |  | **N** |  | **N** |  | **N** | **24.57** | **N** |
| **18** | **49** | **Female** | **13.5** | **N** |  |  |  |  |  |  |  |  | **N** |  | **N** |  | **N** | **13.5** | **N** |
| **19** | **65** | **Female** | **28.87** | **N** |  |  |  |  |  |  |  |  | **N** |  | **N** |  | **N** | **28.87** | **N** |
| **20** | **77** | **Male** | **16.77** | **N** |  |  |  |  |  |  |  |  | **N** |  | **N** |  | **N** | **16.77** | **N** |
| **21** | **55** | **Female** | **22.17** | **N** |  |  |  |  |  |  |  |  | **N** |  | **N** |  | **N** | **22.17** | **N** |
| **22** | **51** | **Female** | **22.7** | **N** |  |  |  |  |  |  |  |  | **N** |  | **N** |  | **N** | **22.7** | **N** |
| **23** | **73** | **Female** | **8.5** | **Y** |  | **●** | **●** | **●** | **●** | **●** |  |  | **Y** | **Nausea & Vomiting** | **N** |  | **N** | **8.5** | **N** |
| **24** | **54** | **Femail** | **6.33** | **Y** |  |  |  |  |  |  | **●** |  | **Y** | **Hepatic insufficiency** | **N** |  | **N** | **6.33** | **N** |
| **25** | **52** | **Female** | **11.67** | **Y** |  |  | **●** |  |  |  |  |  | **Y** | **Nausea & Vomiting** | **N** |  | **N** | **11.67** | **N** |
| **26** | **63** | **Male** | **2.5** | **Y** | **●** |  |  |  |  |  |  | **●** | **N** |  | **Y** | **Death** | **Y** | **2.5** | **Y** |
| **27** | **69** | **Male** | **1.0** | **N** |  |  |  |  |  |  |  |  | **N** |  | **Y** | **Death** | **Y** | **1.0** | **N** |
| **28** | **69** | **Female** | **6.0** | **Y** | **●** |  |  | **●** |  |  | **●** |  | **N** |  | **Y** | **Hepatic insufficiency** | **N** | **8.6** | **N** |
| **29** | **63** | **Female** | **9.0** | **Y** |  |  | **●** |  |  |  |  |  | **N** |  | **N** |  | **N** | **9.0** | **N** |
| **30** | **56** | **Male** | **6.6** | **Y** | **●** |  |  |  |  |  |  |  | **N** |  | **N** |  | **N** | **6.6** | **N** |
| **31** | **61** | **Male** | **10.33** | **Y** |  |  |  | **●** |  | **●** |  | **●** | **N** |  | **N** |  | **N** | **10.33** | **N** |
| **32** | **54** | **Female** | **6.33** | **Y** | **●** |  | **●** | **●** |  | **●** |  |  | **N** |  | **N** |  | **N** | **6.33** | **N** |
| **33** | **68** | **Female** | **8.75** | **N** |  |  |  |  |  |  |  |  | **N** |  | **N** |  | **N** | **8.75** | **N** |
| **34** | **64** | **Male** | **6.1** | **Y** |  | **●** |  | **●** |  |  |  |  | **N** |  | **N** |  | **N** | **6.1** | **N** |
| **35** | **54** | **Female** | **6.03** | **N** |  |  |  |  |  |  |  |  | **N** |  | **N** |  | **N** | **6.67** | **N** |
| **36** | **52** | **Female** | **7.0** | **Y** |  |  | **●** |  |  | **●** |  |  | **N** |  | **N** |  | **N** | **7.0** | **N** |
