# Supplementary material for: Efficacy and Tolerability of Nintedanib in Idiopathic-Inflammatory-Myopathy-Related Interstitial Lung Disease: A Pilot Study
Source: Front Med (Lausanne). 2021 Feb 3;8:626953. doi: 10.3389/fmed.2021.626953 (PMC7886679; doi:10.3389/fmed.2021.626953)
Supplement: Supplementary file 2 [file Table_2.DOCX]

**Supplementary table 2: Distribution of adverse events**

| **Factors** | **Number of patients (percent)** |  |
| --- | --- | --- |
| **Any adverse event** | **25 (69.4%)** |  |
| **Frequent adverse event in 36 patients** | |  |
| **Diarrhea** | **16(44.4%)** |  |
| **Abdominal pain** | **5 (13.9%)** |  |
| **Nausea & Vomiting** | **6 (16.7%)** |  |
| **Anorexia** | **8 (22.2%)** |  |
| **Weight loss** | **2(5.6%)** |  |
| **Fatigue** | **8(22.2%)** |  |
| **Hepatic insufficiency** | **5 (13.9%)** |  |
| **Cough** | **6(16.7%)** |  |
| **In 9 patients with dosage reduction due to adverse events** | | |
| **Diarrhea** | **5(55.6%)** |  |
| **Abdominal pain** | **2 (22.2%)** |  |
| **Nausea & Vomiting** | **3 (33.3%)** |  |
| **Anorexia** | **3 (33.3%)** |  |
| **Weight loss** | **2 (22.2%)** |  |
| **Fatigue** | **3 (33.3%)** |  |
| **Hepatic insufficiency** | **4 (44.4%)** |  |
| **Cough** | **1 (11.1%)** |  |
| **In 5 patients with therapy discontinuation due to adverse events** | |  |
| **Diarrhea** | **5 (100.0%)** |  |
| **Abdominal pain** | **1 (20.0%)** |  |
| **Nausea & Vomiting** | **1 (20.0%)** |  |
| **Anorexia** | **3 (60.0%)** |  |
| **Weight loss** | **1 (20.0%)** |  |
| **Fatigue** | **1 (20.0%)** |  |
| **Hepatic insufficiency** | **3 (60.0%)** |  |
| **Cough** | **0 (0.0%)** |  |
